# Early life exercise training and inhibition of *apoLpp* mRNA expression to improve age-related arrhythmias and prolong the average lifespan in *Drosophila melanogaster*

**DOI:** 10.18632/aging.204422

**Published:** 2022-12-05

**Authors:** Meng Ding, Qiu Fang Li, Tian Hang Peng, Tong Quan Wang, Han Hui Yan, Chao Tang, Xiao Ya Wang, Yin Guo, Lan Zheng

**Affiliations:** 1 Key Laboratory of Physical Fitness and Exercise Rehabilitation of Hunan Province, Hunan Normal University, Changsha, China

**Keywords:** exercise training, arrhythmias, *Drosophila*, apolipoprotein B, aging

## Abstract

Cardiovascular disease (CVD) places a heavy burden on older patients and the global healthcare system. A large body of evidence suggests that exercise training is essential in preventing and treating cardiovascular disease, but the underlying mechanisms are not well understood. Here, we used the *Drosophila* melanogaster animal model to study the effects of early-life exercise training (Exercise) on the aging heart and lifespan. We found in flies that age-induced arrhythmias are conserved across different genetic backgrounds. The fat body is the primary source of circulating lipoproteins in flies. Inhibition of fat body *apoLpp* (*Drosophila* apoB homolog) demonstrated that low expression of *apoLpp* reduced the development of arrhythmias in aged flies but did not affect average lifespan. At the same time, exercise can also reduce the expression of *apoLpp* mRNA in aged flies and have a protective effect on the heart, which is similar to the inhibition of *apoLpp* mRNA. Although treatment of *UAS-apoLpp^RNAi^* and exercise alone had no significant effect on lifespan, the combination of *UAS-apoLpp^RNAi^* and exercise extended the average lifespan of flies. Therefore, we conclude that *UAS-apoLpp^RNAi^* and exercise are sufficient to resist age-induced arrhythmias, which may be related to the decreased expression of *apoLpp* mRNA, and that *UAS-apoLpp^RNAi^* and exercise have a combined effect on prolonging the average lifespan.

## INTRODUCTION

Cardiovascular disease is the leading cause of death worldwide, and aging is crucial in developing cardiovascular disease [1]. The aging of the myocardium is often accompanied by significant electrophysiological changes that significantly increase the risk of arrhythmias in the elderly [2]. The aging of the cardiovascular system is interconnected with longevity through many pathophysiological mechanisms [3]. In fact, dyslipidemia, hyperglycemia, insulin resistance, and other cardiometabolic diseases share common pathological mechanisms with aging and longevity [3]. ApoB is a secreted glycoprotein with 16 N-linked oligosaccharides associated with the egg yolk protein vitellogenin, and its primary function is to carry lipids [4]. Dyslipidemia caused by aging is related to apolipoprotein B [5, 6]. Excessive concentrations of apolipoprotein B in plasma are risk factors for various cardiovascular and metabolic diseases, such as obesity, diabetes, and atherosclerosis [7]. Conversely, inhibition of apoB can prevent obesity and reduce cardiovascular risk [8, 9]. Furthermore, exercise is the cornerstone of life. Sedentary behavior can cause cardiovascular remodeling, obesity, and even sudden cardiac death, which threatens health [10–12]. However, there is considerable difficulty and complexity in studying cardiac aging due to long life spans and genetic redundancy in mammals. Therefore, we utilized the *Drosophila* model to design this experiment.

*Drosophila* are a well established animal model, and its powerful genetic toolkit and short-lived characteristics are the best choices for studying aging [13]. The heart of *Drosophila* is divided into four chambers separated by small flap-like openings, an organ used for hemolymph circulation [14]. Due to the conserved molecular pathways and various efficient assays, the *Drosophila* heart has proven to be a convenient invertebrate model of heart disease [15]. Several exercise models have been developed in *Drosophila* that recapitulates the characteristics of exercise-generated adaptations that are remarkably similar to those of humans or mammals [16–20]. Insect fat body functions identical to the mammalian liver and adipose tissue and plays a crucial role in energy storage and utilization [21]. In *Drosophila*, the lipoprotein (Lpp) resembles mammalian apoB-containing lipoproteins. Lpp production in the fat body requires Mtp, and the *Drosophila* apoB homolog, apolipoprotein (*apoLpp*) [22]. *apoLpp* is a member of the apoB family, conserved across the animal kingdom [22]. When Lpp is secreted from the fat body, it is subsequently recruited to the gut, where they are further loaded with lipids and transported to other tissues [22]. Previous studies have shown that inhibition of fat body *apoLpp* can substantially reduce whole-body lipid levels in flies fed a standard diet, highlighting the critical contribution of fat body *apoLpp* to whole-body lipid metabolism [23].

The broad benefits of exercise on cardiovascular aging have been recognized [24]. For example, regularly trained rats can prevent aging-induced impairment of mitochondrial function and mitochondria-mediated cardiomyocyte apoptosis [25]. Endurance exercise protects aged *Drosophila* from lipotoxic cardiomyopathy [26]. Despite mounting evidence that regular exercise has preventive and protective effects on cardiac aging, the underlying mechanisms remain poorly understood. ApoB is an important marker of cardiovascular events, and it may be involved in a critical part of healthy aging [27]. However, it is unclear whether the protective effect of exercise on the aging heart is related to apoB.

Using a *Drosophila* exercise model, we reveal the benefits of fat body *apoLpp* inhibition for age-related arrhythmias. Significantly, the protective effect of exercise on the aged heart may be related to the decreased expression of *apoLpp*. Another surprising finding is that exercise and fat body *UAS-apoLpp^RNAi^* have a combined effect on low prolongation of average lifespan in *Drosophila*.

## RESULTS

### Aging drosophila exhibits arrhythmic properties similar to those of mammals

**Figure 1:**
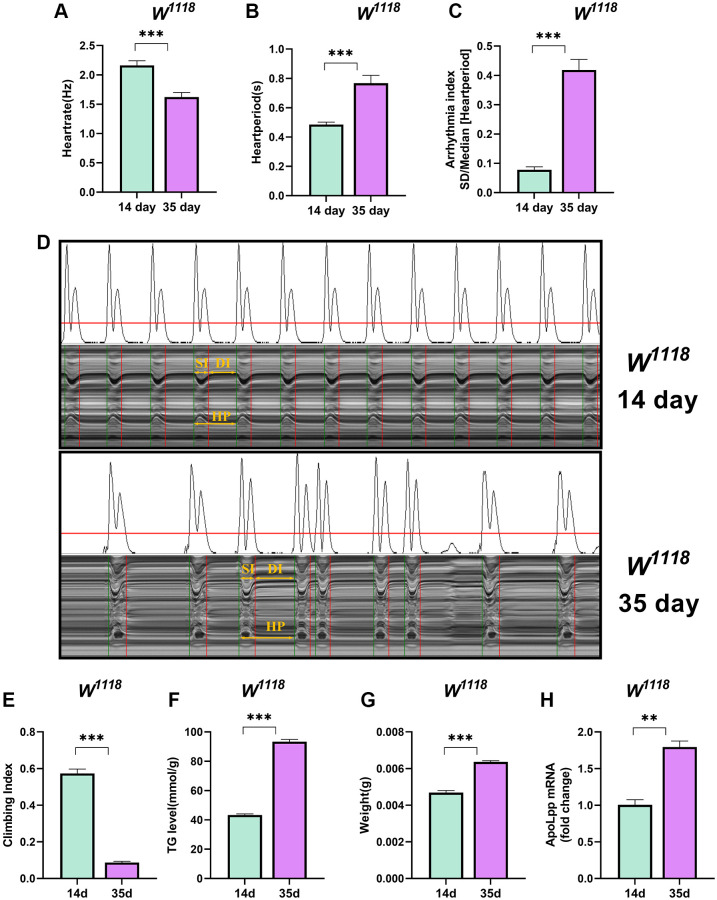
**Older flies are prone to heart rate disorders and disorders of lipid metabolism.** (**A**–**C**) Heart rate, cardiac cycle, and arrhythmia index of 14- and 35-days-old flies in the background of *W^1118^*. *N* = 30. The between-group comparisons were from student *t*-tests. (**D**) M-mode traces (8 s) prepared from high-speed movies of intact flies. The yellow arrows mark the SI, DI, HP, namely the systolic interval, the diastolic interval, and the heart period, respectively. This notation applies to all M-mode traces in this study unless otherwise stated. (**E**) Climbing index of 14- and 35-days-old flies. *N* = 50, see the “Materials and methods” section to calculate the climbing index. (**F**) Whole-body TG levels in 14- and 35-days-old flies. *N* = 5. Error bars represent three independent replicates. (**G**) Body weight of 14- and 35-days-old flies. *N* = 5, values are expressed as the body weight of 5 flies and measured in triplicate. (**H**) Whole-body *apoLpp* mRNA expression levels in 14- and 35-days-old flies. GAPDH was used to normalize these values, *N* = 10. Between-group comparisons were from student *t*-tests. All values except body weight are expressed as mean ± SEM, ^*^p < 0.05, ^**^p < 0.01, ^***^p < 0.001.

Aging is inevitable and the greatest risk factor for cardiovascular disease [28]. The increased incidence of arrhythmias is associated with aging, most commonly from age-induced arrhythmias [29, 30]. We found that the heart rate of 35-days-old flies was significantly lower than that of 14-days-old flies (Figure 1A). In contrast, the heart period and arrhythmia index were significantly higher than 14-days-old flies (Figure 1B–1D). These results may be because aging reduces the spontaneous frequency of heart and increases the arrhythmia index [31].

Aging is often accompanied by decreased physical activity, weight gain, and metabolic disturbances [32]. Indeed, 35-days-old flies had a significant decrease in the climbing index and a significant increase in whole-body triglycerides (TG) levels (Figure 1E, 1F). Furthermore, flies at 35 days weighed significantly more than those at 14 days (Figure 1G). We also found that the expression level of *apoLpp* mRNA was significantly elevated in the whole-body of 35-days-old flies (Figure 1H). A plausible explanation is that older flies have a low ability to scavenge lipids, leading to increased whole-body TG and body weight because these results are similar to humans and mammals [33].

### Inhibition of fat body *apoLpp* mRNA expression rescues age-induced arrhythmias

**Figure 2:**
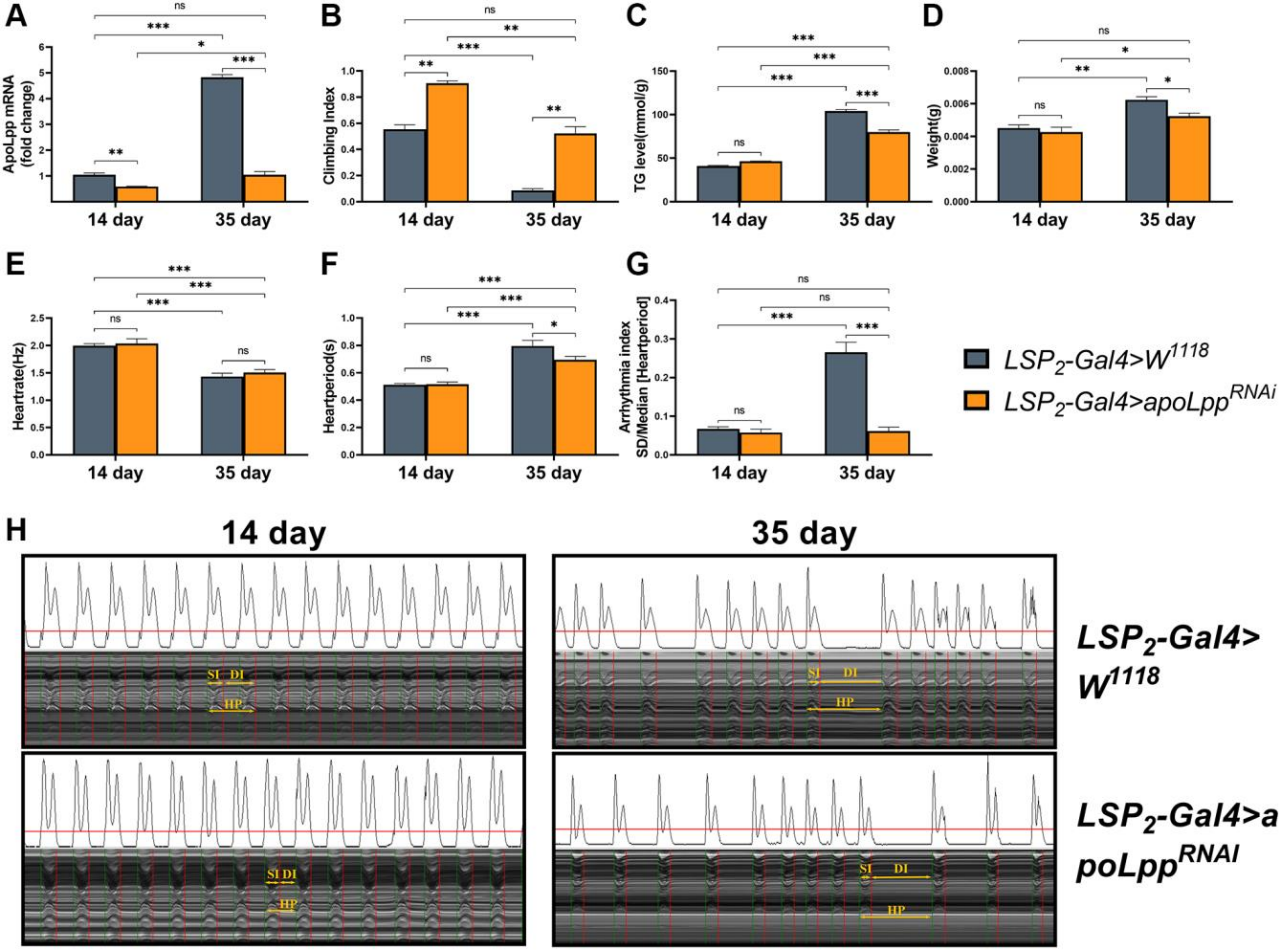
Effects of fat body *UAS-apoLpp^RNAi^* on arrhythmia and lipid metabolism. (**A**) Climbing index of fat body *UAS-apoLpp^RNAi^* in 14- and 35-days-old flies. *N* = 50, see the “Materials and methods” section to calculate the climbing index. (**B**) Whole-body TG levels in 14- and 35-days-old flies by *UAS-apoLpp^RNAi^* in the fat body. *N* = 5. Error bars represent three independent replicates. (**C**) Body weight of 14- and 35-days-old flies with *UAS-apoLpp^RNAi^* in the fat body. *N* = 5, values are expressed as the body weight of 5 flies and measured in triplicate. (**D**) Whole-body *apoLpp* mRNA expression levels in 14- and 35-days-old flies for fat body *UAS-apoLpp^RNAi^*. GAPDH was used to normalize these values, *N* = 10. (**E**–**G**) Heart rate, cardiac cycle, and arrhythmia index of 14- and 35-days-old flies with fat body *UAS-apoLpp^RNAi^*. *N* = 30. (**H**) M-mode traces (8 s) prepared from high-speed movies of intact flies. All *P*-values are from student *t*-tests, all values are expressed as mean ± SEM, ^*^p < 0.05, ^**^p < 0.01, ^***^p < 0.001.

We next asked whether fat body *apoLpp* plays a role in aging. We crossed LPS_2_-Gal4 with *UAS-apoLpp^RNAi^* to generate progeny flies with fat body *apoLpp* targeting knockdown to test this. In addition, the *UAS-apoLpp^RNAi^* strain was backcrossed to the *W^1118^* background for at least eight generations to exclude experimental errors caused by the genetic background. In 14- and 35-days-old flies, inhibition of fat body *apoLpp* reduced the expression of whole body *apoLpp* mRNA by 43.5% and 78.2%, respectively (Figure 2A), indicating that fat body *UAS-apoLpp^RNAi^* was successful. We found that the knockdown of fat body *apoLpp* mRNA reversed the age-induced decrease in the climbing index and restored it to the same level as the control group (Figure 2B). Unexpectedly, inhibition of fat body *apoLpp* mRNA also increased the climbing index of 14-days-old flies beyond controls (Figure 2B). These results remind us of attention deficit hyperactivity disorder (ADHD). In ADHD patients, apoB concentrations are reduced, which may be associated with altered lipoprotein metabolism [34]. Furthermore, we also found that inhibition of fat body *apoLpp* reduced whole-body TG levels and body weight only in 35-days-old flies (Figure 2C, 2D). Although inhibition of fat body *apoLpp* reduced TG levels in 35-days-old flies, it was still higher than in controls (Figure 2C). The above results suggest that inhibition of fat body *apoLpp* is insufficient to counteract age-induced high whole-body TG levels. However, for body weight, inhibition of fat body *apoLpp* could restore body weight to the same level as the control group (Figure 2D). In general, the inhibition of fat body *apoLpp* can resist age-induced low exercise capacity and abnormal lipid metabolism to a certain extent.

Next, an assessment of the fly’s cardiac function found that inhibition of fat body *apoLpp* did not affect heart rate in 14- and 35-days-old flies (Figure 2E). Still, it decreased the heart period in 35-days-old flies, although it did not recover to the same level as the control group (Figure 2F). Similarly, inhibition of fat body *apoLpp* did not affect the arrhythmia index in 14-days-old flies but restored arrhythmias in 35-days-old flies to the same level as controls (Figure 2G, 2H). These data above suggest that inhibition of fat body *apoLpp* can reverse age-induced arrhythmias, which may be mediated by improvements in lipid metabolism.

### Flies fat body *UAS-apoLpp^RNAi^* does not extend lifespan

**Figure 3:**
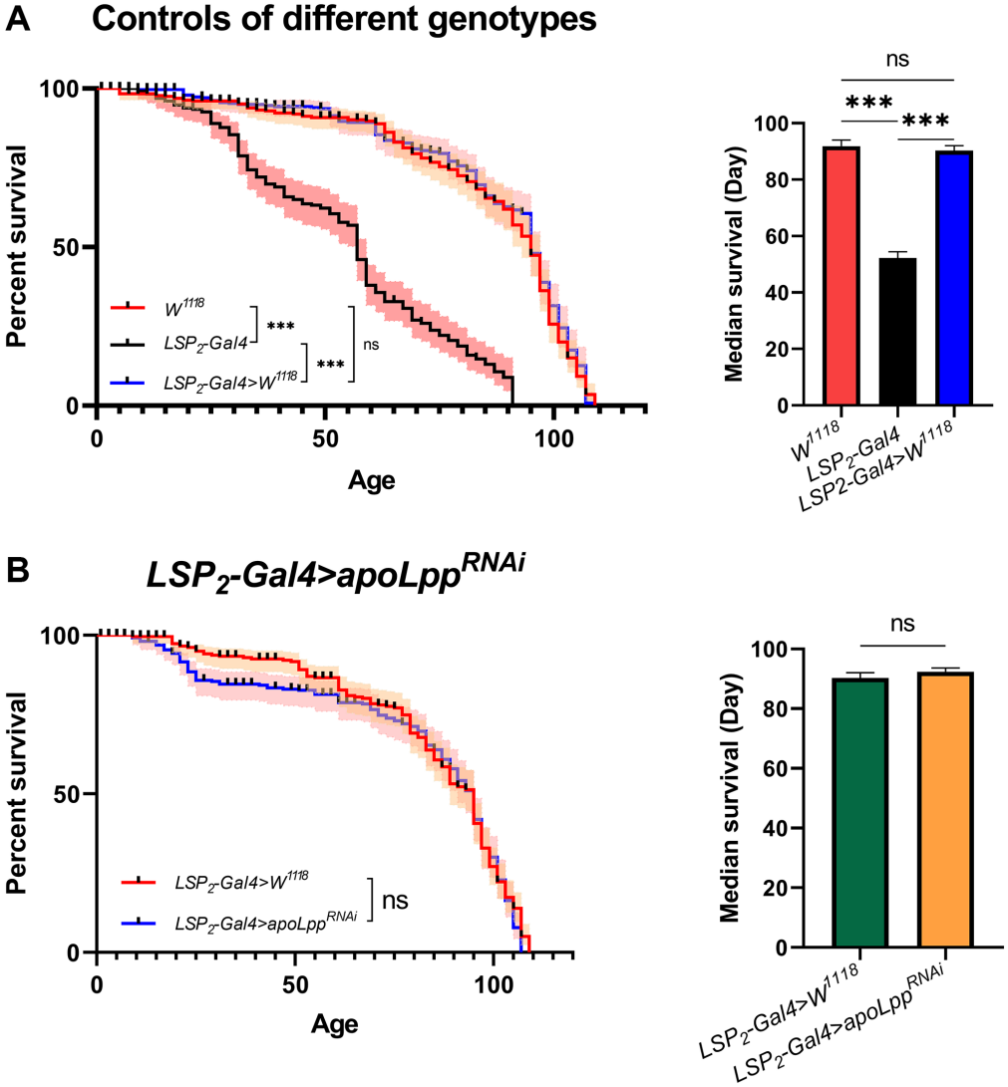
Effects of fat body *UAS-apoLpp^RNAi^* on lifespan. (**A**) Lifespan of different genotypes of flies. On the left is the flies′ survival rate (%), and on the right is the median survival of flies. The sample sizes of *W^1118^*, *LSP2-Gal4*, and *LSP2-Gal4>W^1118^* were 202, 190, and 205. (**B**) Effects of fat body *UAS-apoLpp^RNAi^* on lifespan. On the left is the flies′ survival rate (%), and on the right is the median survival of flies. The sample sizes of *LSP2-Gal4>W^1118^* and *LSP2-Gal4*>*UAS-apoLpp^RNAi^* were 205 and 195. *P*-values for all survival curves were obtained from the log-rank test. For median survival, *P*-values were obtained from student *t*-tests or one-way ANOVA, and all values were expressed as mean ± SEM. ^*^p < 0.05, ^**^p < 0.01, ^***^p < 0.001.

Here, we compared the lifespan of flies in the *W^1118^*, *LSP2-Gal4*, and *LSP2-Gal4>W^1118^* genotypes. We found that flies’ median survival and maximal lifespans in the *LSP2-Gal4* were significantly lower than in the other two backgrounds (Figure 3A). We speculate that this apparent difference may be due to different genotypes, but the reason for this result remains unclear. To interrogate the effect of inhibition of fat body *apoLpp* mRNA on lifespan, we crossed *LSP2-Gal4* with *UAS-apoLpp^RNAi^* flies and used *LSP2-Gal4>W^1118^* as a control. We found that flies’ fat body *UAS-apoLpp^RNAi^* did not prolong the mean lifespan (Figure 3B).

### Exercise saves age-induced arrhythmias but does not prolong median survival

**Figure 4:**
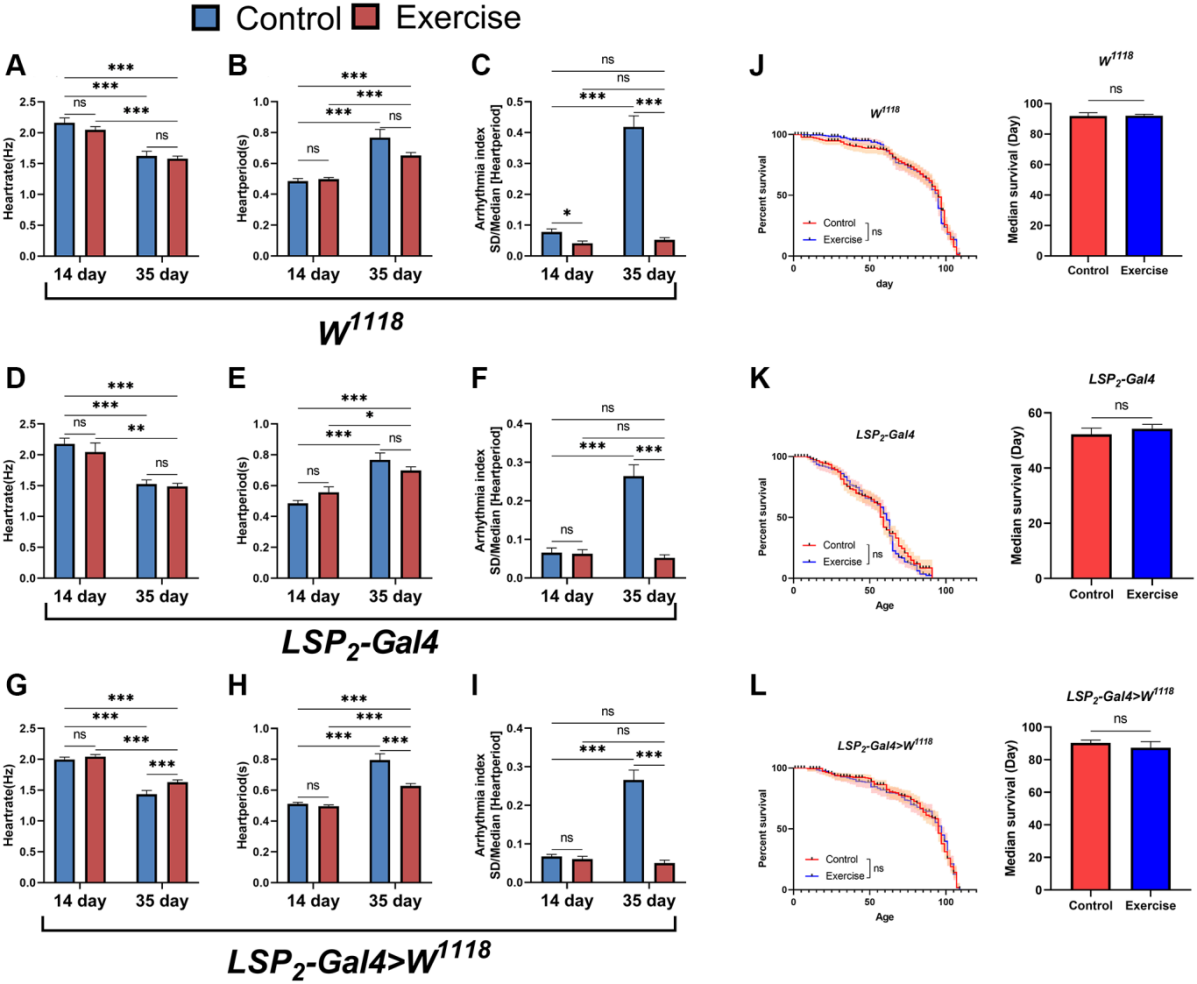
**Effects of exercise on arrhythmias and lifespan.** (**A**–**C**) Heart rate, heart period, and arrhythmia indices in exercise-treated 14- and 35-days-old flies in the *W^1118^*. *N* = 30. (**D**–**F**) Heart rate, heart period, and arrhythmia index in exercise-treated 14- and 35-days-old flies in the *LSP2-Gal4*. *N* = 30. (**G**–**I**) Heart rate, heart period, and arrhythmia index in exercise-treated flies in the *LSP2-Gal4>W^1118^*. *N* = 30. (**J**) The lifespan of exercise-treated 14- and 35-days-old flies in the *W^1118^*. On the left is the fly′s survival rate (%), and on the right is the average lifespan of flies. The sample sizes for Control and exercise were 202 and 210. (**K**) The lifespan of exercise-treated flies in the *LSP2-Gal4*. On the left is the fly′s survival rate (%), and on the right is the average lifespan of flies. The sample sizes for Control and exercise are 190 and 193. (**L**) The lifespan of exercise-treated flies in the *LSP2-Gal4>W^1118^*. On the left is the fly′s survival rate (%), and on the right is the average lifespan of flies. The sample sizes for Control and exercise were 205 and 181. *P*-values for all survival curves were obtained from the log-rank test. All *P*-values except survival curves were from student *t*-tests, and all values are expressed as mean ± SEM. ^*^p < 0.05, ^**^p < 0.01, ^***^p < 0.001.

There is currently solid scientific evidence to support the cardiovascular benefits of regular exercise [24]. However, lack of exercise is a common phenomenon in today’s society. In the elderly, the safety margin of exercise dose decreases with age, which predisposes them to sports injury [35]. Therefore, to circumvent this problem, it will be essential to know whether the effects of exercise can be retained in later life. We performed an exercise intervention in flies using gravity-negative geotaxis. In the *W^1118^* strain, the results showed that exercise-treated 14-days-old and 35-days-old flies had no significant difference in heart rate and heart period compared with their respective controls (Figure 4A, 4B) but reduced arrhythmias index (Figure 4C). In the *LSP2-Gal4* strain, exercise-treated 14- and 35-days-old flies had no significant differences in heart rate and heart period compared with their respective controls (Figure 4D, 4E); only in 35-days-old flies arrhythmic indices were reduced (Figure 4F). In contrast, in the *LSP2-Gal4>W^1118^* line, exercise-treated 14- and 35-days-old flies had increased heart rate and decreased heart period compared with their respective control groups (Figure 4G, 4H); but reduced arrhythmia index only in 35-days-old flies (Figure 4I). Thus, for heart rate and heart period, the reduction in heart rate and the increase in heart period in aged flies were not reversed by exercise, except in the *LSP2-Gal4>W^1118^* strain. Interestingly, although exercise’s effect on reducing arrhythmia index in young flies was unstable across different genotype backgrounds, it was stable in older flies. Therefore, we believe that exercise can effectively reduce the arrhythmia index in aged flies under these three different genotype backgrounds, and the effect of exercise is stable (Supplementary Figure 1). In addition, exercise treatment was more sensitive to *LSP2-Gal4>W^1118^* because aged flies of the *LSP2-Gal4>W^1118^* strain responded positively to exercise in heart rate, heart period, and arrhythmia indices. In addition, we also examined the lifespan of flies. The results showed that exercise did not prolong median survival (Figure 4J–4L). Although we found shorter lifespans in flies in the *LSP2-Gal4*, exercise still failed to extend their average lifespan (Figure 4K).

### Exercise inhibits whole-body *apoLpp* mRNA accumulation in flies and improves age-induced abnormal lipid metabolism

**Figure 5:**
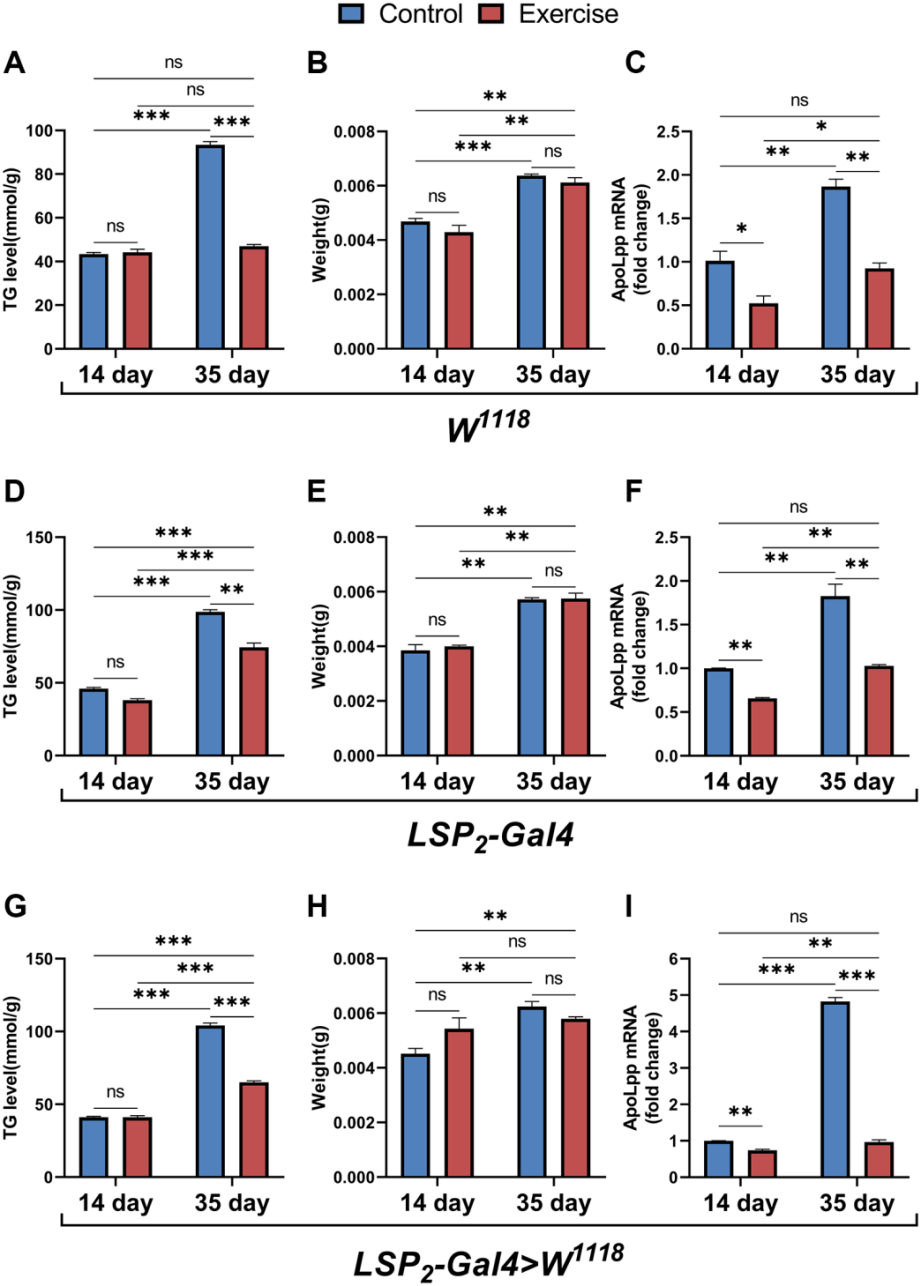
**Effects of ELEL on lipid metabolism in flies under different genotype.** (**A**–**C**) Whole-body TG levels, body weight, and whole-body *apoLpp* mRNA levels in exercise-treated 14- and 35-days-old flies in the *W^1118^*. (**D**–**F**) Whole-body TG levels, body weight, and whole-body *apoLpp* mRNA levels in exercise-treated 14- and 35-days-old flies in the *LSP2-Gal4*. (**G**–**I**) Whole-body TG levels, body weight, and whole-body *apoLpp* mRNA levels in exercise-treated 14- and 35-days-old flies in the *LSP2-Gal4>W^1118^*. To detect whole-body TG, the sample size of all flies was 5, and measured in triplicate. For body weight, values are expressed as the body weight of 5 flies and measured in triplicate. GAPDH was used to normalize these values for whole-body *apoLpp* mRNA expression levels in flies, and *N* = 10. All values are expressed as mean ± SEM, and all *P*-values are from student *t*-tests. ^*^p < 0.05, ^**^p < 0.01, ^***^p < 0.001.

Considering that TG levels are very dependent on genetic background [36], we simultaneously examined the effects of exercise on TG, body weight, and *apoLpp* mRNA in three genotype backgrounds. The results showed that for TG, exercise only reduced TG levels in 35-days-old flies but had no significant effect on 14-days-old flies (Figure 5A, 5D, 5G). Differently, in the W^*1118*^, exercise reduced the age-induced elevation of whole-body TG levels and was comparable to that of the 14-days-old control group (Figure 5A). In contrast, in the *LSP2-Gal4* and *LSP2-Gal4>W^1118^*, exercise did not restore whole-body TG in 35-days-old flies to the same level as in the 14-days-old controls (Figure 5D, 5G). These results indicate that although exercise reduces the whole-body TG of aged flies to different extents under different backgrounds, it can suggest that exercise has an inhibitory effect on triglyceride accumulation in old flies. Regardless of the background, only aging significantly increased body weight, while exercise had no reduced effect (Figure 5B, 5E, 5H). In addition, we also found that exercise significantly reduced the expression of *apoLpp* mRNA in the whole body of 14- and 35-days-old flies under three different genotypes (Figure 5C, 5F, 5I). Interestingly, although exercise reduced whole-body *apoLpp* mRNA expression in 14-days-old flies in three different genotypes, it did not reduce whole-body TG levels (Figure 5A, 5C, 5D, 5F, 5G, 5I). This may be due to the inherently low whole-body TG levels in 14-days-old flies, resulting in a reduced sensitivity of exercise to whole-body TG. In general, these results support that exercise can reduce the expression level of *apoLpp* mRNA whole-body of old flies and improve lipid metabolism.

### Exercise combined with fat body *UAS-apoLpp^RNAi^* improve age-induced arrhythmias and prolongs the average lifespan

**Figure 6:**
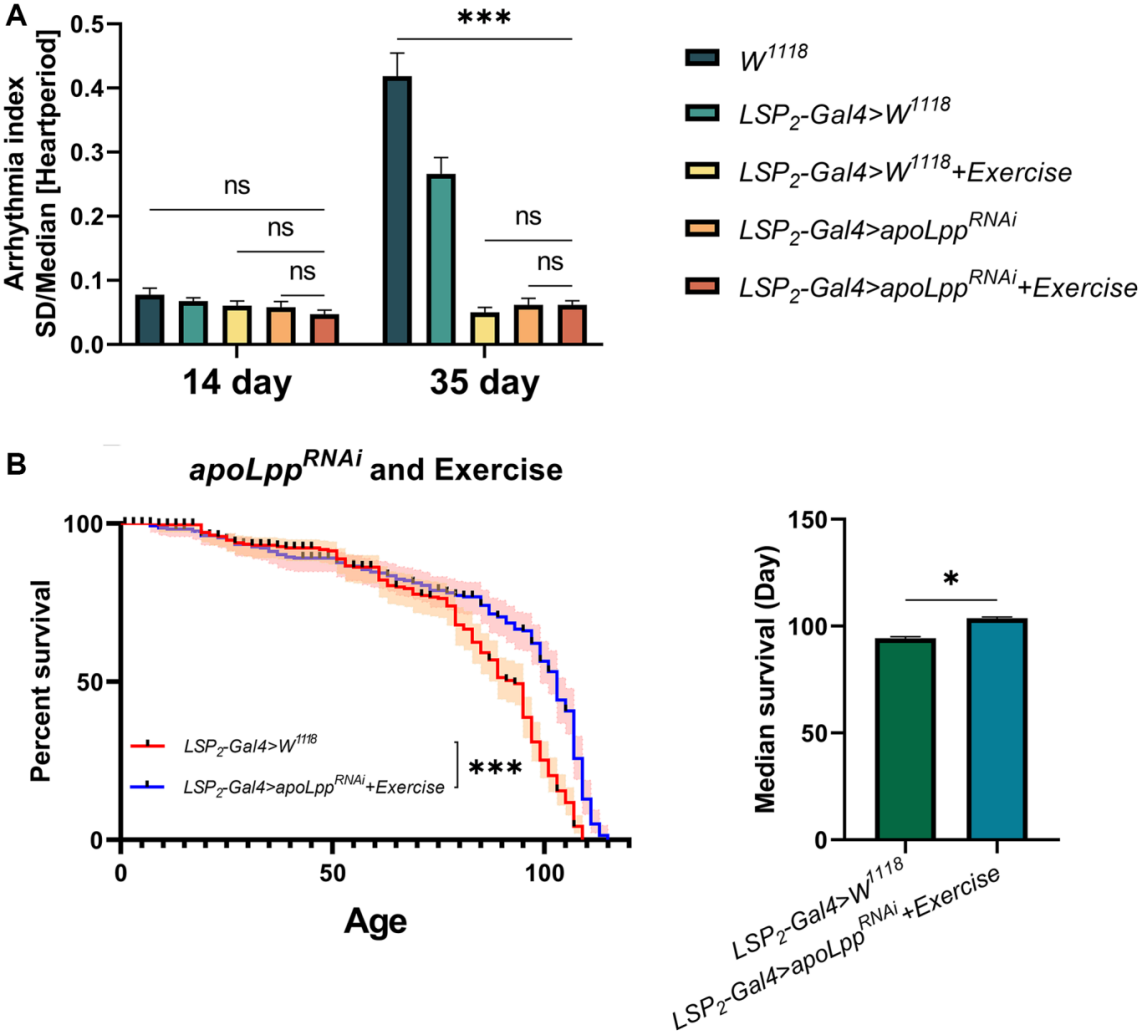
**Effects of exercise combined with fat body *UAS-apoLpp^RNAi^* on lifespan and age-induced arrhythmias in flies.** (**A**) Arrhythmia index in flies under co-action of exercise with fat body *UAS-apoLpp^RNAi^*. *N* = 30. Two-way ANOVA was used for *LSP2-Gal4>W^1118^*, *LSP2-Gal4>W^1118^*+exercise, *LSP2-Gal4*>*UAS-apoLpp^RNAi^*, and *LSP2-Gal4*>*UAS-apoLpp^RNAi^*+exercise followed by post hoc tests using Bonferroni correction. The *P*-values for *W^1118^* and *LSP2-Gal4>W^1118^*+exercise were from student *t*-tests. (**B**) The lifespan of flies under co-action of exercise with fat body *UAS-apoLpp^RNAi^*. On the left is the flies′ survival rate (%), and on the right is the median survival of flies. The sample sizes of *LSP2-Gal4>W^1118^* and *LSP2-Gal4*>*UAS-apoLpp^RNAi^*+exercise were 205 and 183, respectively. The experiment was carried out with 10 technical replicates and three biological replicates. The *P*-values for survival curves were obtained from the log-rank test, and the *P*-values for median survival were obtained from student *t*-tests. ^*^p < 0.05, ^**^p < 0.01, ^***^p < 0.001.

The studies above suggest that although exercise and fat body *UAS-apoLpp^RNAi^* do not prolong the average lifespan of flies, they can improve age-induced arrhythmias. But, exercise-binding fat body *UAS-apoLpp^RNAi^* on lifespan and age-induced arrhythmias in flies is unclear. To elucidate whether the combined effect of exercise and fat body *UAS-apoLpp^RNAi^* could have a more profound impact, we performed exercise on flies with fat body *UAS-apoLpp^RNAi^*. The results showed that in 14-days-old flies, *UAS-apoLpp^RNAi^*+exercise had no significant difference on arrhythmia index (Figure 6A), and it was also not significantly different from *LSP2-Gal4*>*UAS-apoLpp^RNAi^* and *LSP2-Gal4>W^1118^*+exercise (Figure 6A). In 35-days-old flies, although *UAS-apoLpp^RNAi^*+exercise significantly reduced the arrhythmia index, it was also not significantly different from *LSP2-Gal4*>*UAS-apoLpp^RNAi^* and *LSP2-Gal4>W^1118^*+exercise (Figure 6A). These data suggest that although *UAS-apoLpp^RNAi^*+exercise reduced arrhythmia index in aged flies, the combined effect did not confer additional benefits on arrhythmia. Next, we counted the lifespan of the flies. *LSP2-Gal4>W^1118^* was used as a control group. The results showed that *UAS-apoLpp^RNAi^*+exercise extended the median survival of flies by about 9.89% (Figure 6B). In general, these results suggest that the combined effect of *UAS-apoLpp^RNAi^* and exercise has no additional benefit in reducing arrhythmia index in aged flies. Still, it extends the average lifespan of flies.

## DISCUSSION

Aging is a significant cause of cardiovascular disease, including cardiac arrhythmias, fibrillation, and coronary atherosclerosis. The polygenic and multiorgan nature of the aging-induced cardiovascular disease makes it difficult to determine the relative contribution of each of these diseases. We used a *Drosophila* model to simulate aging-induced arrhythmias to elucidate the underlying mechanisms. Because the *Drosophila* model is a simpler system, it can help us examine these complex interactions. *Drosophila* plays an important advantage in age-related heart disease, mainly because of the short life span and easy reproduction characteristics [37]. We exploited the mature genetic toolkit and short-lived characteristics of *Drosophila* to study the crosstalk between apolipoprotein B-mediated lipid metabolism and aging-induced arrhythmias. We found that arrhythmias in aged flies may be associated with elevated apolipoprotein B. Inhibition of *apoLpp* mRNA, a homolog of apolipoprotein B, and early exercise training could reduce the occurrence of age-related arrhythmia risk and promote healthy aging. We provide evidence that old flies exhibit lower spontaneous heart rates and develop arrhythmias, with similar features across different genotypes. In addition, aging also shows increased TG and body weight and decreased climbing ability, which has identical features to age-related abnormal lipid metabolism [33, 38, 39].

Apolipoprotein B is a marker of cardiovascular disease risk and a key molecule in lipid metabolism [40]. Elevated apolipoprotein B (apoB-100) is a common abnormality in insulin-resistant subjects with obesity and type 2 diabetes and increases the risk of cardiovascular disease [41]. In mice, a chronic high-fat diet leads to hepatic apoB accumulation and causes hepatic lipid accumulation and plasma lipid abnormalities [42]. Another study reported that regular exercise in a model of hyperlipidemic mice reduced the increase in apoB induced by HFD and effectively reduced serum triglyceride levels and hepatic lipid accumulation [43]. Recent studies have suggested that apoB-100 may also be associated with the cerebrovascular system, leading to the development of neurodegenerative diseases [44]. However, the relationship between apoB and cardiovascular aging is poorly understood. We investigated the effect of altered fat body apolipoprotein B gene expression on arrhythmias under aging conditions. We found that inhibition of *apoLpp* mRNA expression in the fat body of aged flies reduced the development of age-related arrhythmias. In addition, inhibition of fat body *apoLpp* mRNA also reduced whole-body TG levels and body weight in old flies and improved climbing ability. These results were similar to those in the mouse model, that is, regular exercise effectively reduced the apoB disorder induced by HFD [43]. Recently, studies have begun to reduce apolipoprotein B as an emerging therapy to prevent cardiovascular disease [9, 45]. For example, inhibition of liver apoB mRNA in mice reduced apolipoprotein B concentrations and reduced atherosclerosis [46]. Furthermore, deletion of PCSK9 in mice resulted in decreased lipid and apoB levels and atherosclerotic LDL reduction, and reduced atherosclerosis [47]. Therefore, inhibiting the expression of apolipoprotein B may be an essential mechanism to reduce age-induced arrhythmias. In addition, we also examined the effect of inhibiting the expression of *apoLpp* mRNA on lifespan. We found that flies with different genotypes had large differences in lifespan. Although it is unclear how this difference is caused, fat body *UAS-apoLpp^RNAi^* did not affect flies’ lifespan compared with flies of the same genotypes.

To determine the contribution of exercise to the aging phenotype, we used a *Drosophila* exercise apparatus to simulate exercise [20]. Flies begin exercise training 24 hours after eclosion, which we call early life exercise training or exercise. The results of exercise showed that exercise mainly reduced whole-body TG levels in aged flies but had no effect on body weight. A previous study in rats showed that trained older rats had enlarged hearts and improved cardiac function compared with sedentary older rats [48]. Here, we found that exercise reduced the arrhythmia index in aged flies. The above shows that not only does exercise training in later life provide cardiac benefits, but exercise training early in life can also preserve its benefits into later life. Although studies have shown that maintaining a certain limit of physical activity can reduce the risk of death and achieve the purpose of prolonging life [49], we found that exercise did not prolong the median survival of flies, which may be related to different exercise methods. In addition, exercise also reduced the expression of *apoLpp* mRNA in the whole-body of flies and improved lipid metabolism, and this has similar results to a study in obese mice [43]. Therefore, we believe that exercise reduces age-induced arrhythmias associated with reduced whole-body *apoLpp* mRNA expression.

We present data showing that both exercise and fat body *UAS-apoLpp^RNAi^* can reduce age-related arrhythmias and reduce whole-body TG levels in aged flies. However, exercise and fat body *UAS-apoLpp^RNAi^* treatment alone did not prolong the median survival of flies. Therefore, we also examined whether the combination of exercise and fat body *UAS-apoLpp^RNAi^* could prolong the median survival of flies. The results showed that the combination of exercise and fat body *UAS-apoLpp^RNAi^* extended the median survival of flies. However, this combined effect had no additional benefit for age-related arrhythmias. In a word, our data support that exercise and fat body *UAS-apoLpp^RNAi^* reduce arrhythmias in old flies and that the combination of the two prolongs the median survival of flies.

## MATERIALS AND METHODS

### Fly stocks and maintenance

All fruit flies were *Drosophila melanogaster*. All lines were obtained from the Bloomington *Drosophila* Stock Center: *W^1118^* (BL3605), *LSP2-Gal4* (BL6357), *UAS-apoLpp^RNAi^* (BL33388). All flies were maintained at 25°C, 50% humidity, and a 12-hour light-dark cycle using standard SYA (*Saccharomyces cerevisiae* agar) food. Unless otherwise stated, all used in the experiments were female virgin flies.

### Exercise training

The *Drosophila* exercise training device is designed according to Tower power and Swing boat [16, 18]. As previously, flies were stimulated to actively walk upwards by flipping the vial for exercise [50]. In this study, the flies in the exercise group were placed in the exercise training device within 24 hours after eclosion, working out 2.5 hours a day, 5 days a week with two days off, for a total of two weeks. We call this program Early Life Exercise Training. For ease of presentation, we will refer to Early Life Exercise Training as exercise.

### RT-PCR

Total RNA was extracted using Trizol (Invitrogen, USA) according to the manufacturer’s instructions, and 10 μg of total RNA was synthesized from total RNA using Superscript II reverse transcriptase (Invitrogen, USA) using oligonucleotides (dT). qPCR amplification reactions were performed in triplicate by mixing 1 μl of RT product with 10 μl of SYBR qPCR master mix (TaKaRa, Japan) containing the appropriate PCR primers. Thermal cycling and fluorescence monitoring were performed in an ABI7300 (Applied Biosystems, USA) using the following PCR conditions: (30 s at 95°C, 5 s at 95°C, 30 s at 60°C) × 40. Normalized with GAPDH. The primers used are as follows: GAPDH F: 5′-GCGTCACCTGAAGATCCCAT-3′, R: 5′-GAAGT GGTTCGCCTGGAAGA-3′; *apoLpp* F: 5′-AATTCGC GGATGGTCTGTGT-3′, R: 5′-GCCCCTTAGGGATA GCCTTT-3′.

### Semi-intact *Drosophila* heart preparation and heartbeat analysis

Flies were anesthetized using Fly Nap, the flies were glued thoracic dorsum downward to a Petri dish with petroleum jelly, the head and ventral thorax were rapidly removed, and oxygenated artificial hemolymph (AH) was injected, followed by removal of the ventral abdominal cuticle and all internal organs to expose the ventral canal [51, 52]. A 30-s digital movie of high-speed heartbeats was captured using an EM-CCD high-speed camera at 120–140 fps and recorded using HCImage software (Hamamatsu, Japan). Heart rate, cardiac cycle, arrhythmia index, etc., were precisely quantified using semi-automated optical heartbeat analysis software (available from SOHA, Ocorr, and Bodmer) [51].

### Climbing assay

The negative geotaxis climbing ability test was adapted from a previous method [53]. The climbing device was composed of five 20 cm long glass tubes with an inner diameter of 2.8 cm (sponges were placed at the ends of the tubes to prevent escape but allow air exchange). The sponge plugs at each end of the long glass tube are 2 cm each, allowing 16 cm of climbing space for the flies. The long glass tube is equally divided into 1, 2, 3, and 4 quadrants from bottom to top, and each quadrant is 4 cm. Allow flies to acclimate to the vial for 30 min before assessing negative geotaxis. Negative geotaxis was triggered by tapping the climbing device in rapid succession to drop the flies to the bottom of the bottle. The location of the flies was captured in digital images taken at the end of 10 s after eliciting the behavior. This process was repeated 3 times. 20 flies per tube. The photos were placed in Photoshop for analysis of the climbing index. Climbing index = the number of flies in the fourth quadrant/the total number of flies in the glass bottle.

### Triglyceride and weight assay

The TG content was determined by measuring the absorbance at 510 nm using the Triglyceride (TG) Content Test Kit (mlbio, #ml076637, China) following the manufacturer’s instructions. Take 15 flies, and 1 mL of isopropanol was added and homogenized on ice. Then centrifuge at 12,000 rpm for 10 min at 4°C, and take the supernatant for testing. Triglycerides are hydrolyzed by lipoprotein lipase to glycerol and free fatty acids. Glycerol is then catalyzed by glycerol kinase (GK) to produce glycerol-1-phosphate (G-1-P), which is oxidized by glycerol phosphate oxidase (GPO) to produce hydrogen peroxide (H2O2), which reacts with 4-aminotripyrine to produce a red quinone, with a characteristic absorption peak at 510 nm. Body weight: Flies were weighed using an electronic balance (Uni bloc, AUW220D, Japan). Fifteen Drosophila flies of each genotype were used. Measurements were made by weighing five fruit flies of the same genotype together, and the procedure was repeated three times.

### Lifespan assays

Lifespan assays were mainly performed as described [54]. Flies were reared at a controlled larval density. Flies from one mating were CO_2_ anesthetized, sex-sorted, and transferred to vials (20 flies/vial). Dead flies were counted every 2 days. 10 replicates (= 200 flies) were used for each condition. Female flies were used for all lifespan experiments if not otherwise stated. Survival curves were drawn using GraphPad Prism 6.

### Statistical analysis

Analyses were performed using the Statistical Package for the Social Sciences (SPSS) version 21.0 (SPSS Inc., Chicago, IL, USA) for Windows and graphed using GraphPad Prism 6. Comparisons between different ages were performed using student *t*-tests. Comparisons between different groups of the same age were performed using student *t*-tests (comparison between 2 groups) or one-way ANOVA (comparison among 3 groups). A Bonferroni post hoc test always followed one-way ANOVA. Two-way ANOVA was used to analyze the combined effect of exercise and *UAS-apoLpp^RNAi^*, followed by post hoc testing with Bonferroni correction. *P*-values for survival curves are derived from log-rank. The statistical significance level was set at *p* < 0.05. Data are presented as mean ± SEM unless otherwise stated.

## Supplementary Materials

Supplementary Figure 1

## Abbreviations

Exercise: early-life exercise training
TG: triglycerides
apoB: apolipoprotein B
