# Supplementary material for: Early life exercise training and inhibition of apoLpp mRNA expression to improve age-related arrhythmias and prolong the average lifespan in Drosophila melanogaster
Source: Aging (Albany NY). 2022 Dec 5;14(24):9908–23. doi: 10.18632/aging.204422 (PMC9831727; doi:10.18632/aging.204422)
Supplement: Supplementary Figure 1 [file aging-14-204422-s001.pdf]

SUPPLEMENTARY FIGURE

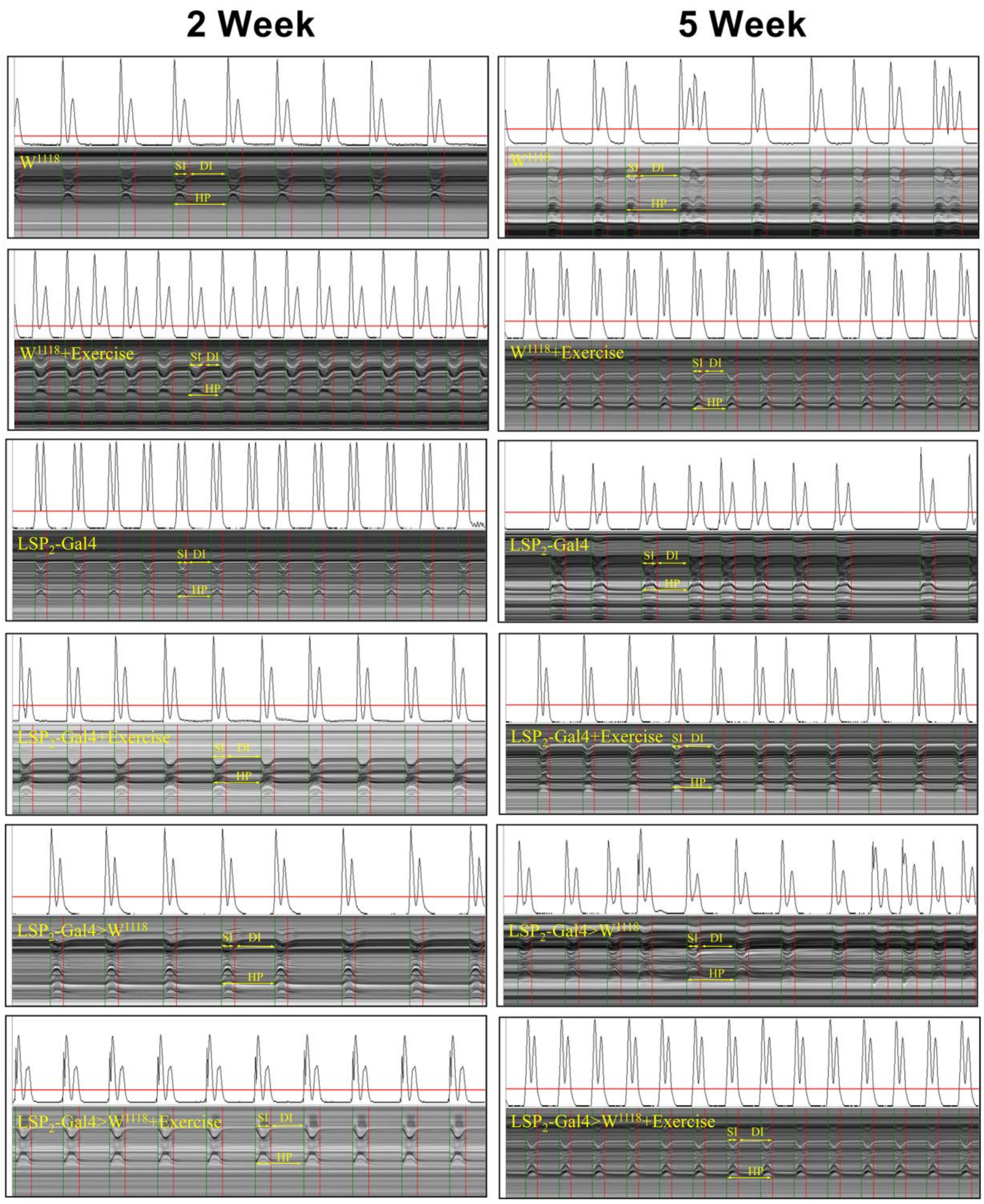

Supplementary Figure 1. M-mode traces (8 s) prepared from high-speed movies of intact flies of 14- and 35-days-old flies.
